# The SHIELD Framework: Advancing Strength-Based Resilience Strategies to Combat Bullying and Cyberbullying in Youth

**DOI:** 10.3390/ijerph22010066

**Published:** 2025-01-07

**Authors:** Stephanie F. Dailey, Rosellen R. Roche

**Affiliations:** 1 School of Education, Counseling Program, George Mason University, Fairfax, VA 22030, USA; 2 School of Medicine, Department of Family Medicine and Population Health, Virginia Commonwealth University, Richmond, VA 23298, USA; rosellen.roche@vcuhealth.org

**Keywords:** bullying, cyberbullying, adolescent mental health, resilience, strength-based framework

## Abstract

Bullying and cyberbullying are critical global issues that significantly affect the mental health and behavioral well-being of youth. This article explores the complex challenges posed by these forms of aggression and introduces a strength-based model for health and mental health professionals to address these issues with impacted youth holistically. Grounded within findings from a scoping review of the literature, the SHIELD framework emphasizes Strengths, Healing, Interventions, Empowerment, Learning, and Development, offering a comprehensive approach for identifying and supporting youth impacted by bullying and cyberbullying. SHIELD emphasizes collaboration among health professionals, schools, families, and communities. By integrating empirical evidence and best practices from school-based approaches to bullying prevention, such as Positive Behavioral Interventions and Support (PBIS) and Social and Emotional Learning (SEL), this framework provides a roadmap for creating safer, more inclusive environments for youth while prioritizing their mental health and overall well-being in the face of bullying and cyberbullying. A case study illustrates the framework’s practical application and future directions are proposed to guide further empirical investigation and stimulate innovative approaches to addressing the complexities of bullying and cyberbullying.

## 1. Introduction

Bullying and cyberbullying represent a pervasive public health concern that affects approximately 20% of youth globally, with 15% of students reporting traditional bullying within their school environment and 30% having at least one cyberbullying experience [1]. Predominately characterized by acts of aggression and power imbalances, these experiences can have a significant adverse impact on youth health and mental health [2,3]. Addressing bullying and cyberbullying requires a nuanced understanding of trauma-informed care that acknowledges both the nature of bullying and the various socio-cultural factors—such as race, gender, and socioeconomic status—that shape how individuals experience victimization [4]. It also requires support systems that extend beyond the school environment, particularly in health and mental health contexts, to ensure holistic and continuous care for impacted youth.

This article reviews current literature and research on bullying and cyberbullying, emphasizing public health implications and evaluating existing prevention and intervention strategies. We introduce the SHIELD framework—Strengths, Healing, Interventions, Empowerment, Learning, and Development—which was developed based on a scoping review of strength-based empirical studies addressing bullying and cyberbullying in children and adolescents. The review identified nine key themes that informed the framework’s integrative approach, tailored for health and mental health professionals. In alignment with many school-based approaches, SHIELD integrates evidence-based principles from the Collaborative for Academic, Social, and Emotional Learning (CASEL) [5] and Positive Behavioral Interventions and Supports (PBIS) [6] to support youth mental health. SHIELD emphasizes youth empowerment by recognizing inherent strengths and focuses on practical ways that youth can better cope with adversity. By promoting self-efficacy, psychosocial support, and long-term strategies to promote resilience, the SHIELD framework provides a comprehensive approach for health and mental health professionals to address and prevent bullying.

## 2. Methods

Development of the SHIELD framework was grounded in a scoping review following the Preferred Reporting Items for Systematic Reviews and Meta-Analyses (PRISMA) guidelines [7]. This approach ensured a comprehensive and systematic identification, analysis, and synthesis of the existing literature on strengths-based approaches to address bullying and cyberbullying among children and adolescents in public health and other related applied settings. The primary objective of the scoping review was to (1) identify key components, principles, and practices of existing strengths-based frameworks to address bullying and cyberbullying for impacted youth, (2) explore the application of these frameworks in public health settings, and (3) establish a theoretical model that health and mental health professionals can use in professional settings.

The methodology for the scoping review was guided by the Arksey and O’Malley [8] framework for scoping reviews, as further refined by Levac et al. [9], which emphasizes: (1) clarifying the research question, (2) conducting a comprehensive search of the literature, (3) selecting relevant studies through iterative screening, (4) charting and synthesizing the data, and (5) reporting results and identifying research gaps. The scoping review addressed the following questions:

1. What are the defining elements of strengths-based frameworks to address bullying and cyberbullying in school and community settings?
2. How are these frameworks applied in public health, particularly in health and mental health care contexts?

### 2.1. Eligibility Criteria and Search Strategy

Inclusion criteria for the scoping review were peer-reviewed, empirical studies, written in English, published between 2013 and 2023 that focused on strengths-based approaches to mitigate bullying and/or cyberbullying among youth aged 6–18. Strengths-based approaches emphasize protective factors (e.g., social support, coping strategies, or emotional regulation) and positive developmental outcomes. Inclusion required studies to report measurable outcomes, interventions, preventative factors, or theoretical frameworks relevant to bullying or cyberbullying. Studies focusing exclusively on deficits or pathology without actual or conceptual references to strengths, opinion pieces without empirical or theoretical basis, and those not addressing bullying or cyberbullying in the specified age group were excluded. Information sources included three academic databases—PubMed, PsycINFO, and Web of Science. Reference lists of key articles were also scanned for additional sources. 

A comprehensive search strategy was developed with input from subject matter experts and a university librarian. The strategy included combinations of keywords and Boolean operators. Keywords included “strengths-based”, “coping mechanisms”, “trauma recovery”, “mental health”, “bullying”, “cyberbullying *”, and “school safety”.

### 2.2. Screening and Selection Process

The screening and selection process for the scoping review adhered to a structured, multi-step methodology to ensure rigor and consistency. The initial search yielded 1426 unique records after duplicates were removed. In Step 1, two reviewers independently screened titles for relevance, resulting in the selection of 210 articles. In Step 2, abstracts were screened, narrowing the dataset to 159 studies that were retrieved for full-text review. During Step 3, full-text screening, 13 studies were excluded based on the predefined eligibility criteria, leaving a final dataset of 143 studies. Discrepancies between reviewers at any stage were resolved through discussion or, when necessary, by consulting a third reviewer to reach consensus. Key information from the selected studies was then systematically extracted using a pre-defined data extraction sheet. This sheet captured essential details such as authorship, year of publication, source, study components, target population, application context, and key findings. This comprehensive and systematic process ensured the inclusion of only relevant, high-quality studies that aligned with the scoping review’s objectives.

### 2.3. Data Synthesis

The synthesis of data followed a structured thematic approach [10] and yielded nine interconnected themes: Resilience and Coping Mechanisms, Early Identification and Prevention, Trauma-Informed and Culturally Relevant Healing, Bullying Literacy, Social and Peer Dynamics, Public Health and Community Integration, Interventions and Program Development, Long-Term Growth, and The Role of Caregivers and Families. These themes, along with their associated subthemes, offer critical insights into the multifaceted nature of bullying and cyberbullying and highlight strengths-based and growth-oriented strategies for improving outcomes for children and adolescents. A summary of the nine themes and subthemes is presented in Table 1.

While these findings highlight effective approaches within school settings, the review revealed a notable gap: the absence of applied models for health and mental health professionals to address the broader implications of bullying and cyberbullying outside of educational contexts. To bridge this gap, the SHIELD framework—Strengths, Healing, Interventions, Empowerment, Learning, and Development—was developed.

**Figure 1:**
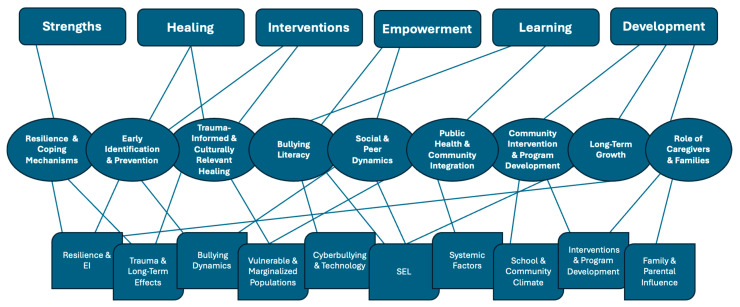
Conceptual map of the SHIELD framework and review themes.

Synthesizing the nine themes and subthemes, the framework provides a structured, strengths-based approach to support impacted youth. Each component reflects key areas identified in the data and offers actionable strategies for health and mental health professionals. Importantly, while bullying and cyberbullying share foundational elements such as aggression and power imbalances, SHIELD’s flexibility allows it to address their unique dynamics. For instance, the anonymity and persistence of cyberbullying require interventions that focus on digital resilience, privacy education, and emotional recovery from online harassment, whereas traditional bullying interventions emphasize peer dynamics, in-person bystander intervention, and fostering safe school environments. This dual applicability ensures SHIELD effectively supports youth across both modalities. Figure 1 maps the interconnected themes and subthemes from the scoping review to the SHIELD components, providing a visual representation of the conceptual map leading to the development of the framework.

The following sections build upon the themes identified in the scoping review, beginning with foundational definitions of bullying and cyberbullying, followed by their impacts and evidence-based strategies for prevention and intervention. Rather than presenting the themes as standalone findings, this article is intentionally structured to guide readers through a progressive understanding of the literature. This approach bridges the gap between research and practice, offering health and mental health professionals accessible, actionable insights to effectively address bullying and cyberbullying across diverse public health settings.

## 3. Understanding Bullying

Bullying is defined as deliberate, aggressive behavior directed toward an individual or group with the intent to harm, intimidate, or humiliate [11]. It is typically marked by a power imbalance, where the aggressor assumes a position of dominance over the victim with the intent of inflicting physical, social, psychological, or emotional harm. Perpetrators often exert control based on differences in social status, physical strength, or group dynamics [12]. Bullying happens repeatedly over time and tends to occur in public settings like school hallways, classrooms, and playgrounds [13]. Such dynamics highlight the importance of early identification and prevention strategies, particularly for health professionals who are well-positioned to recognize and address these behaviors during routine clinical interactions.

The manifestations of bullying vary and often include physical (e.g., pushing or shoving), verbal (e.g., name-calling), and relational forms (e.g., excluding someone from a group or social gathering). Physical bullying involves direct actions such as hitting, kicking, or pushing, while verbal bullying may consist of name-calling, threats, and derogatory remarks. Often more covert, relational bullying involves social exclusion, spreading rumors, or manipulating social relationships to isolate the victim from their peer group [14]. Repeatedly excluding an individual from a lunch table to maintain or establish a social hierarchy is a common example of relational bullying in a school environment. Each of these forms can have serious consequences for the victim, with relational bullying posing unique challenges due to its subtle nature.

Aligned with the Early Identification and Prevention theme from the scoping review, recognizing the diverse forms of bullying early is critical to interrupting harmful behaviors and mitigating their long-term impact. Early detection efforts enable educators, families, and health professionals to implement targeted interventions before harm escalates. Lawrence et al. [15] found that exposure to community violence among African American adolescents led to somatic symptoms, mediated by bullying victimization, highlighting the role of health and mental health professionals in identifying early psychosomatic indicators of bullying-related trauma and implementing trauma-informed care to prevent long-term physical and psychological harm. Cao et al. [16] linked bullying victimization to suicidal ideation, identifying psychological resilience as a partial mediator and emphasizing the need for health professionals to integrate mental health screenings and early interventions that target suicide risk in at-risk youth.

The effects of bullying often extend beyond the immediate context in which the behavior occurs. Impacted youth are at an increased risk of experiencing anxiety, depression, social isolation, and low self-esteem, all of which can significantly hinder academic, social, and emotional development [17]. Maji et al. [18] identified elevated levels of depression, anxiety, stress, and maladaptive coping strategies (e.g., self-blame and rumination) among bullied students compared to their non-bullied peers, underscoring the need for early psychological health screenings to identify at-risk youth and implement targeted interventions, such as teaching adaptive coping strategies and emotional regulation. Prolonged victimization, in extreme cases, can result in self-harm or suicidal ideation, particularly when youth feel trapped in a “downward spiral” of exposure [19] (p. 1). Adverse impacts from chronic bullying can persist into adulthood, leading to long-term mental health concerns, problems forming close interpersonal relationships, and maladaptive social functioning [20]. Jiménez et al. [21] revealed that chronic bullying victimization across developmental stages (e.g., primary school through university) significantly deteriorated emotional well-being, reinforcing the importance of ongoing monitoring and early intervention to disrupt this trajectory and foster resilience. These sequelae reflect the importance of identifying comprehensive interventions for impacted youth and proactive strategies to prevent bullying.

### 3.1. Cyberbullying

Building on the understanding of traditional bullying, this section focuses on cyberbullying—a form of aggression that leverages digital technologies to extend the reach and impact of bullying behaviors. While both traditional bullying and cyberbullying share fundamental characteristics—such as aggression, intent to harm, and repetition—cyberbullying takes place in the digital realm, erasing conventional temporal and spatial boundaries. The covert nature of cyberbullying allows these behaviors to persist continuously and, in many cases, anonymously [22]. Cyberbullying is generally defined as the intentional and repeated infliction of harm through electronic means such as social media platforms, text messages, emails, online forums, and gaming environments [2]. The behaviors involved aim to harass, threaten, humiliate, or intimidate individuals or groups. Common manifestations include direct harassment, wherein victims receive abusive or threatening messages; doxing, where perpetrators share personal or sensitive information without consent to cause harm; cyberstalking, which involves persistent, threatening communications aimed at instilling fear in the victim; and impersonation, where fake accounts are created to damage the victim’s reputation or relationships [23]. Like traditional forms of bullying, cyberbullying includes social exclusion, in which victims are deliberately isolated from online communities or activities [19].

The nature of cyberbullying renders it particularly harmful, as the ease and speed with which harmful content can be disseminated to a widespread audience intensifies its emotional and psychological impact. Marinoni et al. [24] highlight that cyberbullying is often exacerbated by the use of fake profiles, which allow perpetrators to act anonymously, avoiding accountability for their actions. The anonymity and disinhibition afforded by fake identities in online spaces facilitate continuous and covert cyberbullying, as victims often do not know who their aggressors are [22]. Thus, aggressors can operate with fewer constraints, and victims face increased obstacles in finding relief or support [22,25]. Furthermore, the nature of digital communication allows aggressors to target victims at any time, amplifying the sense of inescapability and emotional distress experienced by victims [26]. Addressing these consequences requires comprehensive prevention and intervention strategies tailored to the digital environment, alongside resilience-building measures to support victims and reduce harm [14].

In a large-scale study of over 5000 Chinese college students, Huang et al. [27] found that the anonymous and persistent nature of social media and online gaming enables perpetrators to bully others with fewer constraints, making it particularly difficult for victims to escape such harmful behaviors and seek timely support. Lareki et al. [28] found that victims of cyberbullying involving fake digital identities often experience greater difficulties in identifying their aggressors, making it harder to report or stop the abuse. This inability to trace perpetrators increases victims’ feelings of helplessness and frustration, exacerbating the emotional and psychological impact of cyberbullying. These studies highlight the critical role of public health professionals in identifying covert forms of cyberbullying and implementing early intervention strategies. The anonymity and persistence of online spaces present significant challenges for victims in identifying perpetrators, reporting incidents, and accessing timely support—challenges that public health professionals are uniquely positioned to address through systematic early detection, interdisciplinary collaboration, and targeted intervention efforts.

### 3.2. Prevalence of Bullying and Cyberbullying Among Youth

While the previous section focused on the nature of bullying and cyberbullying, themes from the scoping review emphasize the importance of understanding prevalence and broader public health implications. Globally, approximately one in three students (32%) reports being bullied at least monthly, with significant variations observed regionally [29]. Physical bullying is more prevalent outside Europe and North America, while psychological bullying is more common in these regions [29]. For example, the Health Behaviour in School-aged Children (HBSC) study found that traditional bullying rates in Europe range from 10% in Sweden to 33% in Latvia, with similar variations for cyberbullying in these countries [30]. In East Asia, approximately 30% of youth in Japan and South Korea report bullying, with 10% to 20% reporting cyberbullying [31]. In North America, 20% of students report bullying at school, and 16% have experienced cyberbullying [1].

Occurrences of bullying and cyberbullying vary across countries due to cultural, educational, and social factors that influence victimization trends. For example, Chen et al. [32] found peer victimization rates declining in East Asia but increasing in the U.S. and the U.K., where school disengagement and life satisfaction issues are more pronounced [1]. Similarly, Hong et al. [33] highlighted that parenting styles play a consistent role in bullying victimization across racial and ethnic groups, emphasizing the need for trauma-informed, culturally relevant interventions tailored to each context [13].

Cyberbullying affects a substantial proportion of youth, with variations in prevalence rates reported across studies. For instance, the National Center for Education Statistics (NCES) indicates that 16% of students have experienced cyberbullying within the past year [34], while the Cyberbullying Research Center (CRC) reports that 36.5% of students aged 12 to 17 have encountered cyberbullying at some point [35]. The Centers for Disease Control and Prevention (CDC) highlights that approximately 15.7% of high school students report being electronically bullied in the past year [36]. These variations likely stem from differences in sample populations, methodologies, definitions, and cultural, educational, and social factors shaping victimization trends. Despite these discrepancies, the consensus remains that cyberbullying is a pervasive issue with significant psychological and emotional impacts on youth [1,29,34]. For health and mental health professionals, these statistics reinforce the importance of interventions that address the unique dynamics of cyberbullying, including its persistence, anonymity, and the broader cultural and social factors that influence victimization.

### 3.3. Impact of Bullying and Cyberbullying on Mental and Physical Health

In addition to the contextual influences that shape bullying and cyberbullying, the psychological impact on youth is significant, leading to increased risks of anxiety, depression, social isolation, and low self-esteem. The scoping review highlighted resilience and coping mechanisms as essential for addressing the mental and physical health impacts of bullying and cyberbullying. This section explores these impacts, providing a foundation for health and mental health professionals to leverage these insights to develop targeted interventions that address both immediate and long-term consequences of bullying and cyberbullying.

Victims of bullying and cyberbullying experience a heightened risk of developing mental health issues such as depression and anxiety [17]. These adverse outcomes correlate with the frequency and duration of exposure to aggression [24]. Persistent bullying significantly increases the likelihood of suicidal ideation and attempts [37,38]. While traditional bullying and cyberbullying both correlate with adverse mental health outcomes, cyberbullying has a broader range of influence. Victims of cyberbullying report higher levels of anxiety and emotional distress compared to those experiencing traditional bullying, primarily due to the omnipresent nature of digital harassment and the increased exposure through social media and other platforms [39]. The anonymity inherent in cyberbullying leaves victims feeling more vulnerable [12,19,26]. Such findings emphasize the critical role of trauma-informed and culturally relevant healing practices in clinical and community-based settings, as identified in the scoping review.

Cross-national research illustrates how socio-cultural factors shape the presentation of symptoms and associated mental health impacts. In Asian countries, such as Japan and South Korea, cultural values of shame and honor may intensify psychological effects, leading to elevated rates of depression and social withdrawal [31]. Conversely, in Western contexts, bullying often involves identity-based victimization targeting, for example, race, sexual identity, or gender affiliation [40]. These variations point to the need for culturally informed interventions that consider the specific socio-cultural context of impacted youth [41].

#### 3.3.1. Behavioral Difficulties

Victims of bullying and cyberbullying often exhibit a range of behavioral difficulties. These behaviors can be both direct reactions to the emotional distress caused by victimization and coping mechanisms employed to deal with the sustained aggression. Aggressive behavior is a common outcome, with some affected youth turning to aggression themselves, either as a means of defending against further bullying or to express psychological pain. Studies indicate that bullying victims are more likely to engage in aggressive or delinquent behaviors compared to their non-bullied peers [26].

Substance abuse is often used as a coping strategy for affected youth. Research from North America and Europe indicates that adolescents who have been bullied or cyberbullied are at increased risk of using alcohol, tobacco, or illicit drugs as a means of escaping emotional turmoil [22]. Truancy and poor academic performance are other common outcomes, as victims may avoid school environments to escape bullying, leading to disengagement from academic activities [17].

Finally, bullying and cyberbullying can have a significant effect on the interpersonal relationships of victims. Social withdrawal, a common consequence of emotional distress in youth, can lead to relational problems with peers and family. Impacted youth may find it difficult to trust others and fear further victimization or rejection, leading to isolation and difficulty forming close relationships. It follows that these challenges perpetuate social marginalization and emotional and behavioral problems [23].

#### 3.3.2. Physical Health

Chronic stress associated with sustained bullying has been linked to immune system dysregulation and inflammatory responses, increasing the risk of developing cardiovascular diseases and metabolic syndrome later in life [42]. Victims frequently experience somatic symptoms, including headaches, stomachaches, and musculoskeletal pain, due to the physical toll that emotional distress imposes on the body [43]. Tian et al. [44] found that adolescents subjected to bullying often report muscle tension, particularly in the neck and shoulders. Another common physical consequence is sleep disruption and poor sleep quality, including insomnia, sleep apnea, and nightmares, all of which exacerbate mental health issues like depression and anxiety [39].

The relationship between physical activity and bullying victimization is also concerning. Adolescents who experience body-shaming or weight-based bullying are less likely to engage in physical exercise, increasing their risk of obesity and related health issues [45]. Victims may also develop disordered eating behaviors in response to the stress of bullying [46]. Arseneault [47] found that victims are more likely to suffer from chronic pain, gastrointestinal disorders, and premature aging. Collectively, these findings highlight the importance of addressing the mental and physical health impact of bullying through comprehensive, holistic interventions.

## 4. Addressing Bullying and Cyberbullying in Educational and Community Settings

Bullying and cyberbullying are pervasive public health issues that disrupt the academic, social, and emotional development of youth. Evidence from the scoping review underscores the critical role of schools and communities in shaping peer dynamics, social hierarchies, and behavioral patterns, making them essential intervention points. Such behaviors disrupt individual student trajectories and influence overall school climate, fostering environments marked by fear, insecurity, and disengagement [17,48]. While school-based programs and theoretical frameworks have advanced understanding of bullying dynamics, significant gaps remain in addressing these challenges beyond educational settings.

Educational institutions face unique challenges in addressing cyberbullying due to its persistence in virtual spaces where school authorities have limited oversight [1]. This ongoing threat, extending beyond school hours, hinders effective intervention, as victims often feel unable to escape harassment or seek help [19]. Additionally, the review identifies a lack of frameworks tailored for health and mental health professionals, who frequently address the broader systemic impacts of bullying and cyberbullying in community and clinical settings. These findings highlight the necessity for comprehensive, evidence-based approaches that protect students, build resilience, and address the broader systemic factors perpetuating these behaviors.

Existing interventions, while effective in educational contexts, often fail to tackle systemic and community-level factors. By examining theoretical frameworks and prevention strategies—primarily developed for school environments—this section explores opportunities to adapt and expand these efforts to diverse public health and clinical contexts. Drawing on the Social and Peer Dynamics, Public Health and Community Integration, Bullying Literacy, and Resilience and Coping Mechanisms themes identified in the scoping review, this section highlights key theoretical frameworks, including Social Learning Theory and Ecological Systems Theory, alongside prevention strategies such as school-based programs, cyberbullying awareness campaigns, and community-based initiatives. These insights provide a comprehensive understanding of current approaches and lay the groundwork for developing integrative frameworks that meet the diverse needs of impacted youth beyond school settings.

### 4.1. Current Theoretical Frameworks for Addressing Bullying and Cyberbullying

Theoretical frameworks are crucial in understanding bullying and cyberbullying and shaping effective interventions. Over the years, various theories have been adapted to explain the dynamics of bullying behaviors, each offering unique insights into the cause, effect, and potential solutions. These frameworks range from individual psychological theories to broader social and ecological models, each contributing to a comprehensive understanding of bullying and cyberbullying. The theoretical frameworks explored in this section provide foundational insights into the dynamics of bullying and cyberbullying, aligning with the Interventions and Program Development theme from the review.

One of the foundational models for understanding bullying is Social Learning Theory, introduced by Bandura [49]. According to Social Learning Theory, bullying is a learned behavior, primarily through observing and imitating others in social contexts, such as family environments, peer groups, or media. In this theory, both traditional and cyberbullying behaviors are reinforced by positive outcomes (e.g., gaining power or social status) or by the lack of negative consequences, which perpetuates aggressive behavior. This suggests that interventions should focus on altering the social environment that reinforces bullying through peer education and positive role modeling. Although not developed specifically for bullying, Social Learning Theory informs peer-led interventions aimed at reducing bullying by encouraging positive role modeling and educating youth on the impact of observed behaviors. It also highlights the importance of early identification and prevention programs by detecting environments where bullying behaviors are learned and reinforced.

Another widely utilized framework is Ecological Systems Theory, proposed by Bronfenbrenner [50], which provides a valuable lens for understanding bullying and cyberbullying as behaviors influenced by multiple layers of the social environment, ranging from individual characteristics to broader societal factors. While originally a broader framework, it has been effectively applied to explore how personal factors, family dynamics, and cultural norms shape bullying behaviors. The theory suggests that bullying is not only the result of personal factors, such as aggression or low self-esteem, but also shaped by the microsystem (e.g., family, peer groups), the mesosystem (e.g., interactions between these microsystems), and the macrosystem (e.g., cultural and societal norms) [48]. Ecological Systems Theory emphasizes the need for multi-level interventions that involve schools, families, and communities to create environments that discourage bullying behavior [51]. By addressing these interconnected systems, Ecological Systems Theory underscores the value of public health and community integration, advocating for targeted, multi-level strategies that are sustainable, culturally relevant, and designed to build resilience and empower youth through coordinated efforts across families, schools, and broader societal networks. This multi-level perspective underscores the importance of Public Health and Community Integration—a major theme from the scoping review—in designing interventions that extend beyond educational settings.

The Theory of Planned Behavior, developed by Ajzen [52], has also been applied to bullying and cyberbullying, suggesting that behavior is shaped by three key factors: attitudes toward the behavior, subjective norms (i.e., perceived social pressure), and perceived behavioral control. In the context of bullying, this theory posits that individuals are more likely to engage in bullying if they have positive attitudes toward aggression, if bullying is socially accepted in their peer group, and if they believe they can engage in such behavior without significant consequences [4,17]. Interventions focus on changing social norms around bullying and increasing accountability for aggressive behaviors, thereby reducing the likelihood of bullying. By addressing attitudes and norms, this theory contributes to long-term growth by fostering a shift toward prosocial values and behaviors that extend beyond immediate intervention efforts. It also enhances bullying literacy by promoting awareness and understanding of how individual and collective beliefs influence bullying dynamics, equipping youth with the knowledge and skills to navigate and challenge harmful social pressures.

Lastly, Moral Disengagement Theory, developed by Bandura [53], explains how individuals justify unethical behavior by distancing themselves from the ethical implications of their choices. Bullies may use moral justification (e.g., viewing their actions as a form of punishment or retribution), euphemistic labeling (e.g., calling bullying “just a joke”), or displacement of responsibility (e.g., blaming the victim or attributing their behavior to peer pressure) to rationalize their actions. Interventions focus on understanding the ethical implications of one’s choices, encouraging individuals to take responsibility for their actions, and understanding the harm caused by one’s actions and behaviors [26]. In practice, this theory informs trauma-informed and culturally relevant healing by addressing the cognitive mechanisms that allow individuals to rationalize harmful behaviors, providing a foundation for interventions that promote accountability and empathy while considering the diverse cultural contexts that shape these justifications [4]. Additionally, it supports long-term growth in youth by fostering ethical decision-making as a key skill for resilience and recovery.

#### Limitations of Current Theoretical Frameworks

Theoretical frameworks provide valuable insights into the mechanisms and dynamics of bullying and cyberbullying, forming a foundation for understanding etiology and treatment. However, contemporary research highlights the limitations of these models, especially in addressing the evolving nature of bullying in digital contexts and the socio-cultural factors that shape individual experiences. As such, the absence of frameworks tailored to health and mental health professionals represents a significant gap in the literature. Addressing this gap requires an integrative approach that bridges theory and practice to empower professionals in diverse settings.

Interventions that solely target aggressive behaviors without considering the broader socio-emotional needs of youth have demonstrated limited success [13]. Haines et al. [4] found that entrenched racial divisions fueled bullying victimization, with tensions between White students and Indigenous youth reflecting racism and colonial legacies. Participants also described gendered stereotypes, such as Indigenous girls being labeled as more aggressive, while White students’ socioeconomic privilege reinforced exclusion. These findings underscore the importance of integrative frameworks which address the intersections of identity, culture, and context by promoting resilience, empowerment, and healing through culturally responsive and inclusive strategies.

Recent studies also emphasize the need for holistic approaches prioritizing youth empowerment and positive development rather than merely focusing on harm reduction [6]. Aligned with the theme of Long-Term Growth, these approaches equip youth with skills and strategies that not only address current challenges but also promote resilience, self-efficacy, and emotional well-being into adulthood. Current research provides compelling evidence for the need for holistic approaches to address bullying. For example, Reid et al. [54] found that while childhood bullying victimization is associated with increased anxiety and depression, protective factors like perceived family support significantly buffer these risks during critical transitions, such as the first year of college. This highlights the importance of fostering supportive social environments to promote resilience and positive adjustment rather than merely mitigating harm. Sims-Schouten and Edwards [55] critique narrow resilience frameworks rooted in self-reliance, noting that such approaches often neglect the long-term consequences of bullying and the systemic factors shaping resilience. They call for a more multidimensional understanding that integrates individual empowerment with structural and cultural supports, emphasizing the role of broader social systems. Collectively, these findings reinforce the need for public health-driven frameworks that involve health and mental health professionals in creating comprehensive, community-based strategies. Such approaches prioritize resilience-building and long-term well-being, ensuring youth are supported across individual, familial, and systemic contexts.

### 4.2. Current Prevention Strategies for Bullying and Cyberbullying

Building on insights from theoretical frameworks, current prevention strategies have made significant strides in addressing bullying and cyberbullying. These approaches, spanning school-based programs, cyberbullying awareness campaigns, parental education, and community-based initiatives, reflect the growing emphasis on holistic and multi-level strength-based interventions aimed at increasing youth resilience to combat bullying and cyberbullying. The prevention strategies discussed in this section draw on several themes identified in the scoping review, including the themes of Early Identification and Prevention, Social and Peer Dynamics, and Role of Caregivers and Families. These strategies highlight the importance of multi-level, resilience-oriented approaches for health and mental health professionals.

#### 4.2.1. School-Based Anti-Bullying Programs

School-based programs, as highlighted under the Bullying and Cyberbullying Literacy and Social and Peer Dynamics themes, play a pivotal role in equipping students with the skills to navigate bullying and foster supportive peer environments. School-based anti-bullying programs, as reflected in the Interventions and Program Development theme, emphasize iterative approaches that adapt to emerging needs, combining evidence-based practices with real-time feedback from educators and students. For instance, one of the most widely researched programs is the Olweus Bullying Prevention Program [56] that promotes understanding and awareness of the impact of bullying behaviors [11]. Another is the KiVa program [57], a research-based program developed in Finland that focuses on preventing bullying by promoting empathy and positive student behavior and has curriculum focused on all members of the school community. The Second Step Program [58], also curriculum-based, is a social-emotional learning (SEL) program aimed at reducing bullying by teaching children SEL skills, including empathy and problem-solving, with resources for students, educators, and families. While these are just a few examples, all school-based programs encourage empathy, prosocial behavior, and bystander intervention and many demonstrate high levels of efficacy. For example, the KiVa program has been shown to reduce victimization rates by up to 20% in participating schools [59], and the Olweus Bullying Prevention Program has been found to decrease bullying by 20–23% and victimization by 17–20% [60].

There is a growing emphasis on whole-school approaches, where anti-bullying policies are embedded in school culture, engaging students, staff, and administrators to promote respect and empathy. When compared to modular approaches, extant literature demonstrates that schools implementing whole-school anti-bullying programs significantly reduce bullying behaviors [59]. For instance, Bullying Prevention in Positive Behavioral Interventions and Supports (BP-PBIS) is integrated into the broader framework of Positive Behavioral Interventions and Supports (PBIS), promoting positive behaviors and preventing bullying at all school levels [6]. Positive Behavioral Interventions and Supports (PBIS) is a tiered framework designed to promote positive behavior and reduce disciplinary issues through proactive, evidence-based strategies. PBIS emphasizes creating supportive environments by teaching and reinforcing clear behavioral expectations across all school contexts. At the universal level, PBIS fosters inclusivity and respect, while targeted interventions provide additional support for at-risk students. Programs like Bullying Prevention in Positive Behavioral Interventions and Supports (BP-PBIS) integrate anti-bullying strategies within the broader PBIS framework, addressing bullying behaviors systematically and consistently. Gaffney et al. [59] found that PBIS not only reduces bullying incidents but also improves school climate by promoting respect, empathy, and shared accountability among students and staff.

The scalability and adaptability of PBIS makes it a powerful tool for embedding resilience-building principles into whole-school approaches [61]. For example, the Steps to Respect Program seeks to create a comprehensive anti-bullying culture by involving all stakeholders—students, staff, and parents—in promoting respect and establishing anti-bullying norms [62,63]. This evidence-based program emphasizes empathy-building, effective communication, and bystander intervention, fostering a respectful and inclusive school environment. The Good Behavior Game, another PBIS-based program, has been found to improve classroom behavior and emotional regulation skills, contributing to a decrease in bullying incidents [64]. While not directly part of PBIS, these programs complement whole-school approaches by integrating principles that align with broader frameworks like PBIS and SEL, further enhancing the overall impact of anti-bullying efforts.

The SEL approaches mentioned above have gained prominence for their focus on cultivating supportive environments that enable students to manage peer relationships and reduce aggressive behavior. The Collaborative for Academic, Social, and Emotional Learning (CASEL) framework is a foundational approach in SEL, emphasizing the development of five core competencies: self-awareness, self-management, social awareness, relationship skills, and responsible decision-making [5]. These competencies aim to foster supportive school environments that reduce aggression and enhance prosocial behavior. CASEL’s systemic implementation in schools has been associated with improved academic performance, better peer relationships, and reductions in bullying behaviors [62]. For instance, integrating SEL into daily school practices through CASEL-aligned curricula equips students with the tools to navigate social challenges, build empathy, and engage in constructive conflict resolution. By addressing both individual and systemic factors, CASEL contributes to the creation of school cultures that prioritize emotional well-being and community belonging, aligning closely with resilience-focused interventions. The Promoting Alternative Thinking Strategies (PATHS) program, recognized by CASEL as an evidence-based SEL curriculum, emphasizes emotional awareness, self-regulation, and social problem-solving, aligning closely with core social-emotional learning competencies to foster prosocial behavior and reduce aggressive tendencies in students [64].

A meta-analysis by Taylor et al. [62] found that school-based SEL programs significantly enhance students’ social skills and reduce problem behaviors, including bullying. Similarly, Nickerson et al. [65] demonstrated that students’ perceptions of social-emotional learning (SEL) instruction were inversely related to bullying and victimization, with effects mediated by improved SEL skills. Horner et al. [66] highlighted that implementing PBIS reduces problem behaviors and improves academic performance through proactive, evidence-based strategies. Bradshaw et al. [67] further emphasized that schools adopting PBIS experienced decreased bullying incidents and enhanced perceptions of school safety. In a randomized controlled trial, Waasdorp et al. [68] found that children in schools implementing school-wide PBIS (SWPBIS) displayed lower rates of bullying and peer rejection compared to those in non-SWPBIS schools, with the strongest effects observed in students exposed to SWPBIS at younger ages.

While these frameworks demonstrate efficacy in addressing bullying within school settings, their focus is primarily confined to the educational context. Research highlights the need to extend bullying prevention efforts beyond schools to involve families and communities. For instance, Lester et al. [69] found that engaging parents in school-wide bullying prevention programs significantly enhances their effectiveness. Parents influence children’s involvement in bullying by modeling positive social behavior, offering guidance on responding to bullying, and encouraging help-seeking behaviors. Additionally, the U.S. Department of Health and Human Services [70] emphasizes that bullying prevention requires a community-wide effort, involving families, community organizations, and other stakeholders to create a consistent and supportive environment for children. These findings underscore the need for a more integrative approach that engages educators, public health professionals, mental health practitioners, and community members in addressing the multifaceted challenges of bullying and cyberbullying.

#### 4.2.2. Cyberbullying Awareness Campaigns

As digital interactions among youth continue to rise, cyberbullying awareness campaigns have become a vital part of prevention. These initiatives, aligned with the Public Health and Community Integration theme identified in the scoping review, emphasize the importance of digital literacy and community-wide collaboration to address and mitigate online harassment. These initiatives target students, educators, and families and focus on the risks associated with online communication and tools to recognize and address cyberbullying. A key component of these campaigns is digital literacy education, which teaches students how to navigate online spaces safely. Digital resilience is being integrated into these awareness efforts, empowering youth to recover from negative online experiences and develop coping mechanisms [71,72]. For example, findings from Evans and Smokowski [73] highlight the importance of educational programs that encourage prosocial bystander actions, social support, and community engagement to reduce bullying. See Elsaesser et al. [74], Tozzo et al. [72], and Tao et al. [71] for studies that demonstrate the effectiveness of these campaigns in reducing online harassment.

#### 4.2.3. Parental Education and Involvement

Aligned with the Role of Caregivers and Families theme, recent studies underscore the vital role of parental education programs in helping caregivers recognize signs of bullying and ways to communicate with their children more effectively [74]. These programs promote collaboration between caregivers, schools, and health professionals, creating a cohesive student support system. Tao et al. [71] suggest that regular discussions at home about online behavior help children develop critical thinking skills regarding their digital interactions. Wong et al. [75] found that workshops focused on the emotional and behavioral impacts of bullying significantly enhance caregivers’ understanding, leading to improved communication with their children. Chen et al. [76] concluded that actively engaged caregiving is associated with lower rates of bullying. The authors also note that parental involvement in school-based initiatives enhances the effectiveness of anti-bullying programs. Kwan et al. [1] further highlights the importance of parental involvement, demonstrating that caregivers who monitor their children’s online activity are more likely to engage in meaningful conversations about cyberbullying. Finally, caregiver involvement is also important in therapeutic settings, such as group therapy, where caregivers learn to recognize bullying signs and support effective coping strategies [77]. Collectively, these findings highlight the potential for health and mental health professionals to play a more central role in prevention and intervention efforts by leveraging their access to diverse populations and their ability to implement trauma-informed and resilience-focused practices.

#### 4.2.4. Community-Based Programs

Community-based anti-bullying programs represent a key component of current prevention strategies for bullying and cyberbullying, complementing school-based initiatives, awareness campaigns, and parental education [78]. These programs align with the Public Health and Community Integration theme by building cohesive networks of support that engage families, schools, and health professionals. Their focus is on creating safe environments for youth through education, intervention, and resources tailored to the social and cultural contexts of the communities they serve. The scoping review emphasized how these programs, when guided by trauma-informed and culturally relevant practices, bridge educational settings, families, and public health systems to comprehensively address bullying.

A key aspect of these initiatives is their recognition of the social dynamics and cultural contexts that influence bullying. For example, the LET’s CONNECT (LC) mentorship program exemplifies the positive youth development framework by pairing adolescents with mentors to facilitate social activities and community engagement [78]. Two studies by King et al. [78,79] demonstrated modest improvements in social connectedness, self-esteem, and depression among participants, despite challenges such as resource constraints. This underscores the potential of mentorship-based approaches as a valuable intervention to support youth facing interpersonal challenges.

Health providers and mental health professionals also play a vital role in these community efforts, addressing both the physical and emotional impacts of bullying. The scoping review highlighted research demonstrating that community-based anti-bullying campaigns integrating mental health screenings and associated services significantly reduce bullying incidents [80]. Findings from Holt et al. [81] and Wong et al. [75] further emphasize that offering mental health resources alongside prevention strategies decreases anxiety and fosters a sense of safety among youth. Additionally, health professionals’ direct access to caregivers uniquely positions them to deliver psychoeducation, equip parents with tools to recognize warning signs, and promote positive coping mechanisms, as highlighted by Chen and Zhu [82].

Beyond individual programs—and not specifically mentioned in the reviewed studies—national and local initiatives amplify the impact of community-based efforts by providing resources and fostering engagement. For instance, the National Bullying Prevention Center (NBPC) provides toolkits to raise awareness and foster safe environments (https://www.pacer.org/bullying/, accessed on 18 December 2024) [83], while The Trevor Project offers training and resources to create supportive spaces for LGBTQ+ youth facing bullying (https://www.thetrevorproject.org, accessed on 18 December 2024) [84]. National campaigns such as Bullying Prevention Month and initiatives like Unity Day encourage communities to embrace inclusivity, kindness, and respect.

## 5. Common Treatment Recommendations and Interventions

Addressing the mental health and behavioral consequences of bullying and cyberbullying requires a multifaceted clinical approach that supports both victims and perpetrators. The interventions discussed in this section align closely with the key themes identified in the scoping review, particularly in addressing early identification, resilience-building, and trauma-informed care. The scoping review highlighted the necessity of multifaceted approaches that integrate individual and systemic strategies, bridging research and practice to effectively address the diverse needs of those impacted by bullying and cyberbullying. These findings reinforce the importance of translating evidence-based practices into actionable interventions that health and mental health professionals can implement within both clinical and community contexts.

Cognitive Behavioral Therapy (CBT), for example, is effective in helping individuals process their experiences and develop healthier coping mechanisms, significantly reducing symptoms of anxiety and depression in youth [85]. Dursun and Akkaya [86] conducted a systematic review of Acceptance and Commitment Therapy (ACT)-based group interventions for adolescents. Findings indicated significant improvements in coping competence, peer bullying, and mental health outcomes, underscoring the effectiveness of trauma-informed group therapies in both preventive and intervention contexts. This highlights the importance of group-based approaches for fostering resilience and healing. Trauma-Focused Cognitive Behavioral Therapy (TF-CBT) addresses the specific needs of children who have faced trauma, integrating cognitive-behavioral principles with trauma-sensitive approaches [87]. A meta-analysis by Cohen et al. [88] demonstrates that TF-CBT significantly reduces psychiatric symptoms in children.

Other common approaches include narrative therapy, which helps individuals reframe experiences and mitigate shame [89]. Group therapy, focusing on collective healing, offers a platform for shared understanding and has been found to significantly improve emotional well-being and reduce feelings of isolation [89]. Art and expressive therapies provide creative outlets for emotional expression [90]. Mindfulness-based interventions promote emotional regulation and resilience [91]. Luo et al. [92], for example, utilized a quantitative survey with 420 Chinese children (Grades 3–6, aged ~9.6 years) to explore the relationship between bullying victimization and sleep disturbances. They found that mindfulness significantly buffered sleep disturbances caused by bullying victimization, particularly among boys. This highlights the value of integrating mindfulness practices into school and clinical settings to mitigate psychological and physical impacts of bullying.

Expanding beyond individual-level interventions, systemic strategies like restorative justice programs, bystander interventions, and SEL approaches underscore the critical role of school and community environments in mitigating bullying and fostering supportive peer relations. Assertiveness training empowers victims to communicate their needs and establish boundaries effectively [93]. António et al. [94] underscores the value of tailored assertiveness training and bystander interventions, showing that positive indirect contact, such as imagined interactions, increases younger adolescents’ intentions to intervene in homophobic bullying, especially among females. By addressing mediators like empathic concern and masculinity/femininity threat, these findings support interventions that empower assertive bystander actions, aligning with SHIELD’s trauma-informed and systemic approach. Restorative justice programs facilitate dialogues among victims, perpetrators, and the school community, promoting empathy and accountability [95]. Hebron et al. [96] conducted semi-structured interviews with teachers, parents, and five students with autism spectrum conditions (ASC) in England. They underscored how school ethos and zero-tolerance policies play a protective role for vulnerable youth. A positive school environment, combined with parent–teacher collaboration, was critical to reducing bullying. This aligns with restorative justice approaches and the need for systemic school-wide strategies to build supportive environments.

Bystander intervention programs empower those who witness bullying to act against it, fostering a culture of shared responsibility and improving the school environment [97]. Wolgast et al. [98] emphasized the importance of social perspective-taking and empathy in reducing antisocial behaviors and promoting defending actions. Similarly, peer support and mentorship initiatives create safe spaces for sharing experiences, while restorative justice programs reinforce empathy and accountability by encouraging constructive dialogues between victims and bullies [97]. Research supports these approaches, as Lei et al. [99] demonstrated that beliefs in a just world can buffer aggressive behaviors, suggesting the value of fostering ethical reasoning and accountability. Thornberg et al. [100] found that lower moral disengagement is associated with greater motivation to defend victims and less pro-bullying behavior, underscoring the role of empathy-driven interventions in transforming bullying dynamics. Palmer et al. [101] found that both children and adolescents evaluated proactive bystanders more favorably than passive bystanders, particularly in stigmatized contexts, underscoring the importance of encouraging intervention behaviors in anti-bullying efforts. These findings informed the Bullying Literacy and Social and Peer Dynamics themes, emphasizing the importance of interventions that empower individuals to build empathy, foster accountability, and establish supportive peer networks to effectively address bullying.

The importance of addressing bullying through interventions that foster peer support, empathy, and resilience is consistently supported in the literature. For instance, Wahid et al. [102] conducted qualitative interviews and focus groups in Nepal, revealing adolescent depression’s connections to bullying, family issues, and peer relationships, with loneliness emerging as a central theme. Similarly, Young et al. [103] highlighted the systemic benefits of social and emotional learning (SEL) approaches in their review of 22 qualitative studies on the schooling experiences of pediatric brain tumor survivors, demonstrating how SEL improves peer relationships and reduces bullying by building empathy and resilience. Additionally, Burford et al. [104] evaluated a 60-min gender diversity workshop, finding significant increases in valuing and understanding gender-diverse peers and high confidence in its ability to reduce bullying behaviors, underscoring the impact of targeted, low-resource interventions.

The studies reviewed exemplify the diverse, evidence-based interventions that informed the development of the SHIELD framework. Collectively, these studies highlighted the necessity of both individual and systemic approaches to bullying, which directly shaped the SHIELD framework’s design. While school-based interventions such as peer and parent training, restorative practices, mindfulness, and SEL programs have demonstrated efficacy, the scoping review preceding SHIELD’s development identified significant gaps in community-based prevention, mitigation, and risk identification. The SHIELD framework addresses these gaps by integrating trauma-informed and culturally relevant strategies that meet the needs of diverse populations and broader contexts. Health and mental health professionals, with their unique access to youth through clinical and community contexts, are the primary focus of the framework, which was developed to address gaps in existing strategies for these professionals to effectively mitigate and respond to bullying and cyberbullying. Their involvement is integral to fostering resilience and facilitating recovery and extending support to families and broader community systems and complements existing school-based interventions.

### The Role of Resilience in Bullying Prevention and Treatment

Resilience plays a pivotal role in mitigating the effects of bullying and cyberbullying, empowering youth to adapt and recover from adversity. Research consistently highlights resilience as both a treatment priority and a preventive measure, bridging school-based interventions with broader public health strategies. SEL programs, which foster emotional awareness, self-regulation, problem-solving skills, and healthy relationships, are foundational to building resilience in educational settings [103]. Gan et al. [105] demonstrated that positive youth development attributes associated with SEL, such as emotional growth and adaptive coping mechanisms, significantly reduced the likelihood of cyberbullying victimization [106]. Bradshaw et al. [67] revealed that a lack of peer connections increased vulnerability to bullying, further reinforcing the importance of fostering healthy relationships as a critical component of resilience-building efforts [37]. In both online and offline contexts, these programs enhance relationships, cultivate a sense of belonging, and enable youth to navigate bullying challenges effectively [106].

Friendships, a cornerstone of social-emotional development, are instrumental in these processes, offering critical emotional support and buffering against the negative effects of bullying and victimization. Fraser-Smith et al. [107] found that youth associated friendships with feelings of safety and happiness, viewing their absence as a source of isolation. Similarly, Young et al. [103] emphasized the importance of fostering friendships for pediatric brain tumor survivors returning to school, highlighting their role in improving social experiences, resilience, and reducing bullying. Bradshaw et al. [67] further demonstrated the protective nature of strong peer connections, showing how they reduce vulnerability to multiple forms of bullying and its adverse outcomes.

The role of parents and caregivers in fostering resilience also cannot be overstated. Hinduja and Patchin [108] found that positive parenting practices, such as warmth, structure, and autonomy support, were associated with reductions in bullying and cyberbullying behaviors, while rejection and chaos increased risks. Similarly, Wright and Wachs [109] report that parental social support buffered the negative impacts of cyberbullying, reducing health complaints, suicidal ideation, and self-harm. These findings, aligned with the Role of Caregivers and Families theme, suggest that building supportive home environments strengthens youths’ ability to form and maintain positive peer relationships, amplifying the resilience-building effects of friendships.

The scoping review identified resilience as a central thread connecting all nine themes foundational to the development of the SHIELD framework. Resilience and Coping Mechanisms equip youth with practical skills to navigate adversity, while Bullying Literacy emphasizes understanding bullying dynamics and fostering emotional intelligence. The themes of Social and Peer Dynamics and Public Health and Community Integration highlight the importance of cultivating healthy relationships and embedding trauma-informed and culturally relevant anti-bullying efforts within broader systems. The theme of Early Identification and Prevention focuses on proactive recognition of risk factors, while Trauma-Informed and Culturally Relevant Healing addresses the unique needs of individuals. Collaborative efforts reflected in Interventions and Program Development and the Role of Caregivers and Families further ensure comprehensive and sustainable systemic support. Finally, Long-Term Growth underscores the need for ongoing evaluation and adaptation to promote recovery, resilience, and enduring well-being. Together, these themes position resilience as both a critical pathway to recovery and a foundation for systemic, sustainable change.

Building on these interconnected themes and the pivotal role of resilience in mitigating the impacts of bullying and cyberbullying, the SHIELD framework was developed to offer a comprehensive, evidence-informed approach for health and mental health professionals. By intentionally integrating resilience-building principles and addressing the diverse social, emotional, and systemic factors identified in the scoping review, SHIELD aims to bridge gaps in current practices and provide a unified framework for prevention, intervention, and recovery. The following section introduces the SHIELD framework, detailing its core components, theoretical underpinnings, and practical applications. Designed to operationalize resilience and promote sustainable growth, SHIELD serves as a roadmap for health and mental health providers to address bullying and cyberbullying through inclusive, trauma-informed, and culturally relevant strategies that prioritize well-being and long-term growth.

## 6. The SHIELD Framework

The SHIELD framework presents a comprehensive approach to addressing the complex challenges associated with bullying and cyberbullying among youth. It equips health and mental health professionals with practical strategies grounded in its core components: Strengths (S), Healing (H), Interventions (H), Empowerment (E), Learning (L), and Development (D). The SHIELD framework emphasizes the need for individualization and flexibility so health professionals can tailor their interventions to meet the diverse needs of each patient. Recognizing that everyone possesses inherent strengths and abilities, it aims to provide youth with tools to navigate adversity while fostering increased self-efficacy and long-term well-being [110].

SHIELD builds on the foundational insights of Social Learning Theory, Ecological Systems Theory, the Theory of Planned Behavior, and Moral Disengagement Theory by highlighting the intersections of individual behaviors, social systems, and environmental factors. For instance, the emphasis on peer dynamics and role modeling in Social Learning Theory aligns with SHIELD’s focus on interventions and learning. Similarly, Ecological Systems Theory underscores the importance of addressing multi-level influences through learning and development.

The SHIELD framework also draws heavily on foundational frameworks like CASEL and PBIS, incorporating their emphasis on social-emotional competencies, systemic behavioral support, and multi-tiered intervention strategies. By integrating these evidence-based approaches, SHIELD advances their application in addressing bullying and cyberbullying, with a specific focus on resilience, empowerment, and culturally responsive practices tailored to health and mental health professionals. By synthesizing these theoretical perspectives and applied approaches with a scoping review of current literature, SHIELD offers a strength-based resilience framework designed to equip health and mental health professionals with effective tools for supporting youth.

Understanding the SHIELD acronym is essential to grasp the framework’s comprehensive strategy against bullying and cyberbullying. The first component, Strengths (S), emphasizes the need for health and mental health professionals to identify and leverage each patient’s unique strengths. Professionals are encouraged to facilitate strength-based discussions that empower youth to recognize their abilities and how these strengths can be harnessed to address bullying-related challenges. For instance, during a counseling session, a mental health professional might use a technique called ’strengths spotting’, inviting youth to recount a recent experience where they felt proud or accomplished [111]. If a patient shares a story about helping a friend in need, a counselor can highlight this as an expression of prosocial support and empathy, reinforcing the patient’s ability to influence others positively [2]. This validates the youth’s feelings, fosters a sense of empowerment, and encourages them to apply this strength in challenging situations.

During a wellness exam, a physician can engage the youth by asking open-ended questions about their interests and hobbies, emphasizing how participation in sports or arts can promote teamwork and emotional expression [106]. These strength-based interactions validate the youth’s identity, enhance self-esteem, and help them develop adaptive coping strategies to mitigate mental health impacts [112]. By focusing on genuine engagement and authentic conversations, health and mental health professionals can ensure that youth feel supported and understood, ultimately opening the door to promote the second component—Healing.

Healing (H) emphasizes the importance of providing youth with a safe space to process their bullying experiences. Creating a supportive environment enables professionals to assist youth in developing healthier coping mechanisms and fostering resilience [91]. Similarly, positioning healing after strengths enhances youth’s self-efficacy in confronting adversity. Mental health counselors, for example, who identify self-awareness as a strength of impacted youth, can incorporate mindfulness techniques, such as focused breathing or guided imagery, to help patients manage anxiety and emotional distress associated with processing their bullying experiences [91]. During wellness check-ups, physicians can foster a safe and confidential environment by initiating discussions about emotional well-being with questions such as, “How have things been going for you at school? If you’re comfortable, we can talk about any challenges you’ve been facing with friends, classmates, or teachers, but if you prefer to discuss something else first, that’s okay too”. Such an approach ensures that the patient feels secure in sharing sensitive information and empowers them to choose what they wish to discuss. This approach creates a safe space for healing and aligns with trauma-informed care principles.

Trauma-informed practices allow practitioners to create environments where youth can securely navigate their emotions and experiences [13,112]. Furthermore, inquiring about emotional well-being and bullying from a trauma-informed perspective validates the experiences of affected youth, conveying that their feelings are valid and deserve attention [4,112].

The third component, Interventions (I), emphasizes the role of health and mental health professionals in implementing evidence-based strategies to address bullying and cyberbullying. Importantly, interventions leverage impacted youth’s unique Strengths (S) and Healing (H) needs. Custom-tailored interventions, such as CBT, can help victims reframe negative thoughts and develop adaptive coping skills, leading to clinically significant reductions in symptoms of anxiety and emotional distress [85]. For cyberbullying, interventions focus on equipping youth with the ability to report abusive behaviors through platform-specific tools, fostering digital literacy, and addressing the emotional toll of online harassment, such as anxiety stemming from the persistent visibility of harmful content. Integrating healing-centered practices, including client-centered approaches, ensures that interventions are sensitive to individual experiences [113]. Incorporating mindfulness practices into therapeutic sessions can bolster emotional regulation, helping youth manage stress effectively and fostering a sense of safety and trust within the therapeutic relationship [91].

Health professionals can also play a key role in fostering resilience and healing by providing resources for or facilitating peer support groups, which reduce feelings of isolation and build a sense of community [89,107]. Another option for healing is psychoeducation for youth and their families to enhance awareness of bullying dynamics and improve communication, thereby strengthening family support systems and facilitating the healing process [114]. Importantly, professionals must recognize when to refer youth to specialized services, such as trauma-informed counseling, to address persistent mental health issues or self-harm risks. Collaborating with local mental health services ensures a comprehensive support network that empowers youth to navigate the complexities of bullying and cyberbullying, promoting lasting resilience and well-being [81].

The fourth component, Empowerment (E), focuses on equipping youth with essential skills and confidence to advocate for themselves and others against live and digital bullying. Health and mental health professionals play a pivotal role in this process by providing targeted training through education, role-playing, and community involvement that emphasizes self-advocacy and assertive communication. In the context of cyberbullying, empowerment includes teaching assertive digital communication, guiding youth on advocating for themselves in online spaces, and fostering collective responsibility among peers to intervene in harmful digital interactions. For instance, role-playing scenarios within therapeutic settings allow youth to practice how to assertively address bullying behaviors or seek assistance when feeling threatened. This is supported by research linking assertiveness with improved self-esteem and reduced victimization rates for youth who have experienced bullying [2,112].

Professionals can also educate youth about their rights and available resources in both school and online contexts, empowering them to take proactive steps when encountering bullying and instilling a sense of agency. Mental health professionals, for example, can facilitate discussions about safe online behaviors, emphasizing the importance of reporting harmful content and seeking support from trusted adults or peers [3]. Health professionals can encourage parents and caregivers to support their children in understanding and asserting their rights regarding bullying and cyberbullying by offering workshops or resources promoting constructive conversations about online safety and ways to enhance support at home [38].

Fostering Empowerment (E) also involves youth advocating for their peers and cultivating a culture of collective responsibility and community resilience. By incorporating bystander intervention training into broader empowerment initiatives, professionals can equip youth with the skills to confront bullying behaviors effectively. This proactive approach enables youth to address negative actions and creates an environment where such behaviors are less tolerated [95]. Ultimately, this holistic approach enhances resilience and well-being, fostering a supportive atmosphere where youth can navigate bullying-related challenges.

The fifth component, Learning (L), reflects the need for health and mental health professionals to provide educational opportunities that deepen youth’s understanding of bullying dynamics, coping mechanisms, and social skills. Engaging all youth in SEL-focused educational programs about bullying and cyberbullying has been effective in reducing bullying behaviors while promoting mental health [106]. Workshops focused on empathy, emotional regulation, and conflict resolution equip youth with essential skills for navigating social interactions. Digital literacy education, a key component for addressing cyberbullying, emphasizes responsible online behaviors, understanding privacy settings, and identifying and reporting harmful content to trusted adults. Integrating digital literacy education into these programs teaches youth responsible online behavior, the implications of cyberbullying, and the importance of reporting harmful activities and seeking help from trusted adults [26]. By emphasizing these programs in treatment, health professionals empower youth to respond effectively to such challenges and cultivate a supportive peer environment by fostering a comprehensive understanding of live and digital bullying.

The sixth and final component, Development (D), emphasizes the importance of continuous knowledge growth for youth and their caregivers and health professionals in addressing bullying and cyberbullying. Schools should update students and caregivers annually regarding the latest statistics and strategies for addressing bullying and cyberbullying. Psychoeducational materials should include information on how to identify bullying and cyberbullying, as well as contemporary manifestations of these behaviors.

For professionals, ongoing professional development is essential to remain informed about the latest research, trauma-informed care practices, and resilience-building strategies, enhancing their capacity to support youth effectively [114]. By integrating new knowledge through training and workshops, professionals improve their skills and contribute to educating youth. Development fosters a collaborative approach, uniting health providers, educators, and community organizations to create comprehensive support networks. While indirect, this holistic approach builds long-term resilience in youth by ensuring professionals can not only identify bullying and cyberbullying but are prepared to guide the recovery process.

### 6.1. Case Study

The following case study illustrates the application of the SHIELD framework in supporting the mental and emotional well-being of a 7th-grade male student, John (pseudonym), who is experiencing cyberbullying. In this example, we explore how the SHIELD framework can be effectively implemented to empower John, address the challenges he faces as a victim of cyberbullying, and sustainable ways to foster his resilience.

Readers should note that while the SHIELD framework is designed to be flexible and applicable to both bullying and cyberbullying, this case study focuses specifically on its application to cyberbullying as an illustrative example. The framework’s adaptability allows it to address both traditional and virtual bullying occurrences. For instance, in cases of traditional bullying, Strengths (S) might emphasize building peer support networks within school or community settings; Healing (H) could involve trauma-informed practices tailored to physical or relational aggression; and Interventions (I) might focus on creating safe, monitored physical environments to disrupt in-person aggression. Similarly, Empowerment (E), Learning (L), and Development (D) can be tailored to address the unique environmental and relational aspects of traditional bullying.

#### 6.1.1. Background

During a routine wellness exam, 12-year-old John presents with physical symptoms, including stomach aches and sleep disturbances. His pediatrician, noticing the psychosomatic nature of these complaints. After building rapport and ensuring John understood this was a safe environment, the pediatrician asks about any stressors or recent changes in his life. John discloses that he has been experiencing persistent online harassment, including derogatory messages and exclusion from social media groups. This harassment includes hurtful messages such as, “You’re a loser for liking that game; nobody wants to hang out with you”, and threats like, “If you keep talking to my friends at school, I’ll tell everyone your secrets and get everyone to ignore you”. John is also no longer invited to participate in group chats or play online games with his friends, intensifying his loneliness. This experience has significantly impacted his self-esteem, leading to emotional distress and affecting his academic performance. Recognizing the need to support John, the healthcare provider adopts the SHIELD framework and engages key stakeholders, including mental health professionals, John’s family, and his broader community.

#### 6.1.2. Strengths (S)

This first component emphasizes recognizing and leveraging John’s inherent strengths. The healthcare provider validates John’s courage in disclosing his experiences and asks, “Who are the people in your life that you feel most comfortable turning to for support?” This Strengths-based inquiry affirms John’s feelings and helps identify his support network. The provider collaborates with John to explore his interests and skills, such as his artistic talent, problem-solving abilities, and resilience in facing challenges. Encouraging John to express himself creatively through art and writing fosters his self-esteem and empowers him to begin healing. Additionally, John’s strong relationships with friends and family can be highlighted as a source of support, emphasizing how these connections can help him navigate his difficulties.

#### 6.1.3. Healing (H)

This second component focuses on Healing emotional wounds caused by bullying. Mental health professionals, referred by John’s pediatrician, provide a safe space for John to express his feelings about the cyberbullying experience. Therapeutic techniques such as mindfulness and cognitive-behavioral strategies are integrated to help John process his emotions, manage anxiety, and build resilience. Consideration is given to John’s unique identity and potential challenges, such as pressure to conform to traditional masculine norms, experiences of racial or ethnic microaggressions or discrimination within his school or community, or difficulties related to neurodiversity (e.g., ADHD, autism) or learning disabilities. Each of these factors could contribute to social exclusion, bullying, or barriers to emotional expression and help-seeking. These inquiries provide a holistic understanding of John’s experiences to guide healing. Finally, by aligning John’s strengths, such as his creativity and problem-solving skills, with healing strategies, professionals encourage him to use artistic expression as a therapeutic outlet. This fosters emotional healing and enhances self-efficacy. Taking these healing factors into consideration, John develops a self-care plan that includes healthy coping mechanisms, such as journaling and physical activity, promoting his physical and emotional well-being.

#### 6.1.4. Interventions (I)

This third component addresses specific Interventions tailored to John’s needs. The healthcare provider collaborates with mental health professionals to implement strategies aimed at creating a more supportive environment. This includes recommending individual therapy sessions where John can engage in role-playing exercises to practice assertive communication when addressing bullying behavior. The provider establishes precise reporting mechanisms, empowering John to seek help when needed. Furthermore, training sessions for bystanders are proposed, educating peers on how to intervene safely and effectively when witnessing cyberbullying.

#### 6.1.5. Empowerment (E)

Empowerment within the SHIELD framework centers on equipping John with the skills, knowledge, and confidence necessary to advocate for themselves and navigate bullying challenges effectively. Grounded in SEL competencies, particularly self-management and relationship skills, the empowerment component aims to enhance self-efficacy and resilience. Empowerment tools, such as role-playing and skill-building activities, are effective tools to reduce victimization and foster resilience [54]. For example, scenario-based training on assertive communication, combined with psychoeducation about digital safety, builds John’s capacity to address cyberbullying constructively. By fostering John’s sense of personal and collective agency—drawing from moral and social development theories—he can develop the self-efficacy and understanding of his rights needed to counter bullying effectively. Research indicates that youth who feel empowered and equipped with these skills are better prepared to address and mitigate the harmful effects of bullying [55].

#### 6.1.6. Learning (L)

This fifth component emphasizes helping youth and their caregivers to learn about their rights and available resources. The healthcare provider provides John and his family with resources, such as in-person and virtual training resources on digital citizenship and online safety. The purpose is to educate John and his family about the importance of reporting harmful online behaviors and understanding personal rights in school and digital contexts. The provider encourages John’s family to engage in these discussions frequently, reinforcing the home–community connection and ensuring consistent messaging about safety and support.

#### 6.1.7. Development (D)

The Development component focuses on the ongoing growth of John and the healthcare professionals involved in his care. The healthcare provider schedules regular follow-up appointments to monitor John’s physical and emotional health, and the mental health professional continues to work with John as necessary. Providers participate in professional development opportunities to stay current with the latest research on bullying prevention, trauma-informed care, and resilience-building. Continuous development of the providers’ skills informs the approach taken with John and current or future patients like him, ensuring that interventions are evidence-based and effective. Again, by focusing on a holistic and comprehensive approach, the providers can share insights with colleagues and enhance the care provided to youth facing similar challenges. Interdisciplinary collaboration with health and mental health professionals, educators, and community organizations further strengthens John’s support network, creating a cohesive approach to addressing bullying and promoting resilience.

### 6.2. Role of Health and Mental Health Professionals

Throughout this process, medical professionals implement the SHIELD framework for John. During follow-up visits, the pediatrician assesses John’s overall health and monitors his psychosomatic symptoms. The provider educates John and his family about the impact of stress on physical health, emphasizing healthy lifestyle choices, including regular exercise and balanced nutrition. Additionally, the provider discusses stress management techniques, such as deep-breathing exercises, to help John cope with anxiety effectively.

Mental health professionals outside the school setting, such as professional counselors, contribute significantly to John’s resilience-building process. These professionals can offer specialized therapeutic interventions tailored to John’s unique circumstances, such as TF-CBT. This approach helps John process his experiences and develop effective coping strategies while facilitating family sessions to enhance communication and understanding within the household. By fostering a collaborative relationship between healthcare providers, a comprehensive support network is established that reinforces the SHIELD framework’s principles of empowerment and resilience.

Implementing the SHIELD framework in John’s case illustrates the effectiveness of a comprehensive, collaborative approach to addressing the challenges of cyberbullying. By focusing on strengths, healing, tailored interventions, education, and ongoing development, health and mental health professionals can empower youth like John to navigate bullying situations effectively, cultivate resilience, and enhance their overall well-being.

## 7. Limitations and Future Directions

Despite its comprehensive design, the SHIELD framework has limitations. First, while its theoretical foundations are drawn from widely used existing theories, such as Social Learning Theory, and frameworks like PBIS and CASEL, these models have inherent variability in their implementation, which may affect the consistency of SHIELD’s application. Future research should develop structured protocols and fidelity measures to ensure SHIELD’s effectiveness while allowing for necessary contextual adaptations. Second, while SHIELD emphasizes healing through trauma-informed, culturally relevant approaches and promotes individualized interventions, these strategies may not sufficiently address the acute psychological needs of impacted youth. Continued utilization of crisis assessment and risk management protocols is critical to ensure immediate safety and stabilization for youth in high-risk situations. Third, SHIELD’s focus on individual-level resilience and empowerment may limit its capacity to address systemic and environmental contributors to bullying and cyberbullying, such as school culture, family dynamics, and community norms. Future research should explore how public health professionals can leverage SHIELD to implement multi-level interventions that address these systemic factors, fostering community-based initiatives and advocating for structural changes that create inclusive and supportive environments. Future research should also explore the application of SHIELD in broader everyday contexts, such as community programs and family settings, to maximize its reach and impact beyond health and mental health professionals.

Finally, the SHIELD framework has yet to undergo comprehensive empirical validation. To establish content validity, outcome effectiveness, and practical utility, a Delphi study is recommended as an initial step. This systematic and iterative method of achieving expert consensus will engage multidisciplinary professionals in bullying prevention, mental health, education, and public health to evaluate SHIELD’s conceptual underpinnings, ensuring its relevance, clarity, and comprehensiveness. Outcome validation should follow through robust pretest–posttest designs with intervention and control groups, establishing relationships between SHIELD’s application and observed outcomes. Key metrics include reductions in bullying and cyberbullying victimization, improvements in resilience, and enhancements in mental health indicators, such as reduced anxiety and depression levels. Employing mixed methods approaches, integrating quantitative measures with qualitative analysis, will provide a comprehensive evaluation of the framework’s impact. Scenario-based testing through qualitative methodologies, such as semi-structured interviews and focus groups, will further explore SHIELD’s feasibility, cultural responsiveness, and contextual relevance. By engaging students, educators, health professionals, and families, these discussions will uncover barriers and facilitators to implementation, guiding iterative refinements and ensuring SHIELD’s adaptability across diverse settings.

## 8. Conclusions

The SHIELD framework presents a comprehensive and innovative approach to addressing the critical issues of bullying and cyberbullying among youth. This framework integrates evidence-based practices that promote resilience and emotional well-being by emphasizing Strengths, Healing, Interventions, Empowerment, Learning, and Development. The escalating prevalence of bullying and cyberbullying, alongside their detrimental effects on mental health, necessitates the implementation of holistic strategies that not only mitigate immediate harm but also foster long-term resilience.

The SHIELD framework encourages a community-wide commitment to creating safe and supportive student environments by incorporating trauma-informed care, social-emotional learning, and collaboration between health professionals, educators, and families. The framework’s integrative nature suggests a natural progression through its key components, guiding practitioners in effectively addressing the complexities of bullying and cyberbullying. This sequential approach encourages practitioners to identify and leverage the strengths of youth first, followed by facilitating healing, implementing targeted interventions, fostering empowerment, enhancing learning, and supporting ongoing development. While it is not a rigid step-by-step process, the SHIELD framework provides a flexible structure that practitioners can adapt based on individual needs, prioritizing aspects like Healing (H) when urgent support is required.

Ultimately, SHIELD serves as a crucial step toward transforming the landscape of bullying prevention, promoting resilience, and enhancing the well-being of youth in an increasingly interconnected world. By recognizing and harnessing individual strengths, facilitating healing processes, and providing targeted interventions, this framework empowers young individuals to navigate the challenges of bullying and cyberbullying effectively, ensuring they have the tools needed to thrive.

## Figures and Tables

**Table 1:** Identified themes and subthemes.

Theme	Subtheme	Description
Resilience and Coping Mechanisms	Resilience and Emotional Intelligence ^1^	Focuses on developing emotional intelligence and utilizing strengths such as reframing and social support to cope.
	Trauma and Long-Term Effects ^1^	Examines how trauma impacts individuals over time and how resilience mitigates and/or moderates adverse effects.
Early Identification and Prevention	Bullying Dynamics^1^	Identifies factors such as power imbalances and risk indicators to detect and address bullying early.
	Resilience and Emotional Intelligence ^1^	Highlights the role of emotional intelligence and resilience as protective factors to identify vulnerabilities.
Trauma-Informed and Culturally Relevant Healing	Trauma and Long-Term Effects ^1^	Investigates the emotional and psychological outcomes of trauma, including IPV and bullying victimization.
	Vulnerable and Marginalized Populations ^1^	Addresses barriers to healing experienced by marginalized groups such as LGBTQIA+ youth and refugee populations.
Bullying Literacy	Cyberbullying and Technology	Explores the role of digital spaces in bullying and how digital literacy can help mitigate victimization.
	Social and Emotional Learning ^1^	Promotes programs that develop skills like empathy, self-awareness, and online safety to prevent bullying.
Social and Peer Dynamics	Bullying Dynamics ^1^	Analyzes social hierarchies and peer relationships that contribute to bullying behaviors and victimization.
	Social and Emotional Learning ^1^	Enhances social skills and healthy peer interactions through SEL initiatives to foster positive dynamics.
Public Health and Community Integration	Vulnerable and Marginalized Populations ^1^	Focuses on systemic barriers and discrimination, aiming to identify supportive environments for marginalized youth.
	Broader Implications and Systemic Factors	Takes a biopsychosocial approach to bullying to inform public health strategies and community policy initiatives.
Interventions and Program Development	School and Community Climate	Promotes school and community programs that create safe, inclusive environments to reduce bullying.
	Interventions and Program Development	Focuses on designing and implementing targeted anti-bullying programs and abuse prevention strategies.
Long-Term Growth	Resilience and Emotional Intelligence ^1^	Strengthens emotional intelligence and resilience to prepare youth for long-term emotional and personal success.
	Social and Emotional Learning ^1^	Equips individuals with lifelong skills in managing relationships, decisions, and social challenges.
Role of Caregivers and Families	Family and Parental Influence	Highlights the role of caregivers in buffering bullying impacts and fostering supportive environments.
	Interventions and Program Development	Emphasizes parental education as part of comprehensive anti-bullying and resilience-building programs.

^1^ Subthemes appearing under multiple themes reflect overlapping areas of focus within the included studies. Subtheme titles were retained during coding, with final definitions refined to demonstrate alignment with the respective major themes.

## Data Availability

The raw data supporting the conclusions of this article will be made available by the authors on request.
